# Exploring the New Horizon of AdipoQ in Obesity-Related Alzheimer’s Dementia

**DOI:** 10.3389/fphys.2020.567678

**Published:** 2021-01-27

**Authors:** Md. Sahab Uddin, Md. Motiar Rahman, Mohammad Abu Sufian, Philippe Jeandet, Ghulam Md. Ashraf, May N. Bin-Jumah, Shaker A. Mousa, Mohamed M. Abdel-Daim, Muhammad Furqan Akhtar, Ammara Saleem, Md. Shah Amran

**Affiliations:** ^1^Department of Pharmacy, Southeast University, Dhaka, Bangladesh; ^2^Pharmakon Neuroscience Research Network, Dhaka, Bangladesh; ^3^Institute of Synthetic Biology, Shenzhen Institute of Advanced Technology (SIAT), Chinese Academy of Sciences (CAS), Shenzhen, China; ^4^Research Unit, Induced Resistance and Plant Bioprotection, EA 4707, SFR Condorcet FR CNRS 3417, Faculty of Sciences, University of Reims Champagne-Ardenne, Reims Cedex, France; ^5^Pre-clinical Research Unit, King Fahd Medical Research Center, King Abdulaziz University, Jeddah, Saudi Arabia; ^6^Department of Medical Laboratory Technology, Faculty of Applied Medical Sciences, King Abdulaziz University, Jeddah, Saudi Arabia; ^7^Department of Biology, College of Science, Princess Nourah bint Abdulrahman University, Riyadh, Saudi Arabia; ^8^Pharmaceutical Research Institute, Albany College of Pharmacy and Health Sciences, New York, NY, United States; ^9^Department of Zoology, College of Science, King Saud University, Riyadh, Saudi Arabia; ^10^Pharmacology Department, Faculty of Veterinary Medicine, Suez Canal University, Ismailia, Egypt; ^11^Riphah Institute of Pharmaceutical Sciences, Riphah International University, Lahore, Pakistan; ^12^Department of Pharmacology, Faculty of Pharmaceutical Sciences, Government College University Faisalabad, Faisalabad, Pakistan; ^13^Department of Pharmaceutical Chemistry, Faculty of Pharmacy, University of Dhaka, Dhaka, Bangladesh

**Keywords:** Alzheimer’s disease, adipokine, AdipoQ, adipocyte dysfunction, obesity

## Abstract

Alzheimer’s disease (AD) is the most common form of dementia, which causes abnormalities in learning, thinking, memory, as well as behavior. Generally, symptoms of AD develop gradually and aggravate over time, and consequently severely interfere with daily activities. Furthermore, obesity is one of the common risk factors for dementia. Dysregulation of adipokine and adipocyte dysfunction are assumed to be accountable for the high risk of obesity in people that develop many related disorders such as AD. Moreover, it has been observed that the dysfunction of adipose is connected with changes in brain metabolism, brain atrophy, cognitive decline, impaired mood, neuroinflammation, impaired insulin signaling, and neuronal dysfunction in people with obesity. Conversely, the pathological mechanisms, as well as the molecular players which are involved in this association, have been unclear until now. In this article, we discuss the impact of adiponectin (AdipoQ) on obesity-related Alzheimer’s dementia.

## Introduction

Alzheimer’s disease (AD) is the most frequent type of dementia among elderly individuals (Kabir et al., 2020a; Uddin et al., 2020d). It is characterized by the presence of two major hallmarks in the brain such as neurofibrillary tangles, predominantly formed by hyperphosphorylated tau proteins, and senile plaques, mainly composed of amyloid-beta (Aβ) peptides (Kabir et al., 2020b; Mamun et al., 2020). The latter hallmark is a major product of the proteolytic cleavage of the amyloid precursor protein (APP) by β- and γ-secretase enzymes (Patterson et al., 2008; Uddin et al., 2020a). A major characteristic of AD is sporadic memory damages related to hippocampal affectation. However, several pathological changes, such as oxidative stress, insulin resistance, neuroinflammation, or mitochondrial damage, associated with obesity also result in AD pathological development (Najem et al., 2014; O’Brien et al., 2017; Ferreira et al., 2018; Uddin and Kabir, 2019).

Copious reports regarding the increase in body fat mass as well as the possibility of suffering from dementia have been published in the past few years (Whitmer et al., 2005; Businaro et al., 2012; Kivimäki et al., 2018). But, their outcomes are disputed and at several times inconclusive. It appears that it is significant to distinguish between being overweight at middle-age and in late-life (Xu et al., 2011), precisely, obesity in middle-aged life and reduction of bodyweight in the preclinical phase typify dementia (Singh-Manoux et al., 2018). Indeed, in a meta-analysis of 21 trials, some authors have revealed that midlife obesity (i.e., <65years) is associated with the development of dementia, but not after 65years (Pedditizi et al., 2016). Another study investigated 1,349,857 individuals from 39 diverse cohorts with body mass index (BMI) data measured at baseline (Kivimäki et al., 2018). This study found that 20years before dementia diagnosis, increased BMI was strongly related to a higher risk of dementia in mid-life. Analyses also demonstrated that this risk was inverted among late-life where an elevated BMI could even be a defensive factor against the risk of dementia (Kivimäki et al., 2018).

Adiponectin (AdipoQ) is a 30 KDa adipokine encoded by the *AdipoQ* gene, mostly produced by adipocytes and is rich in human plasma (Dendana et al., 2018). AdipoQ is recognized to enhance the insulin susceptibility of target organs including muscles and liver, finally controlling fatty acid metabolism and peripheral glucose levels (Hotta et al., 2001; Yamauchi et al., 2001, 2002). Also, being a metabolic controller, AdipoQ is additionally identified for its antioxidant and anti-inflammatory effects (Takemura et al., 2007; Liu et al., 2015b). The central nervous system (CNS) is not relieved from the negative impact of obesity since obesity-related adipose malfunction has been connected to distorted neuroinflammation, brain metabolism, brain atrophy, neuronal dysfunction, cognitive decline, and impaired mood (Luppino et al., 2010; Gustafson, 2012; Arshad et al., 2018). People who suffer from obesity are under higher threat to disease progression in the case of age-linked cognitive failure, mild cognitive impairment (MCI), vascular dementia, and AD (Frisardi et al., 2010). Dysregulation of AdipoQ signaling may disrupt brain homeostasis and increase the risk of cognitive impairment. A better understanding of the impact of AdipoQ in AD is thus crucial. Particularly, restoring typical AdipoQ signaling might establish constructive, disease-modifying therapeutics against AD. Therefore, this article presents recent studies about AdipoQ on obesity-related AD.

## AdipoQ and Brain Targets

AdipoQ was primarily identified in 1995 in 3T3-L1 adipocyte differentiation (Scherer et al., 1995). It is considered as one of the most abundant adipokines taking into account its plasma levels compared to several other hormones (Matsuzawa, 2005; Thundyil et al., 2012; Polito et al., 2018). AdipoQ self-assembles into bigger shapes inducing homotrimers that further self-assemble to construct hexamers or dodecamers. A globular AdipoQ resulting from the degradation of the monomer was additionally recognized (Waki et al., 2003). AdipoQ is largely produced and discharged from adipocytes, but, it is currently well-documented that these adipokines are produced by the liver, placenta, epithelial cells, pituitary cells, osteoblasts, and myocytes (Wilkinson et al., 2007; Psilopanagioti et al., 2009; Thundyil et al., 2012). Remarkably, various reports demonstrated AdipoQ mRNA expression in human pituitary (Psilopanagioti et al., 2009) and chicken diencephalon (Maddineni et al., 2005; Wilkinson et al., 2007). In the human pituitary, AdipoQ might have a role in the discharge of gonadotrophs and somatotrophs (Thundyil et al., 2012). Also, it controls a wide variety of metabolic actions such as the regulation of body-weight, lipid catabolism, glucose regulation, anti-atherogenic process, insulin sensitivity as well as endothelial function (Berg et al., 2002; Okamoto et al., 2002; Stefan and Stumvoll, 2002; Whitehead et al., 2006; Thundyil et al., 2012). Such activities are induced by three diverse receptors, namely T-cadherin (CDH13), AdipoQ receptor 1 (AdipoQR1), and AdipoQ receptor 2 (AdipoQR2), and involve various signaling cascades such as AMP-activated protein kinase (AMPK), peroxisome proliferator-activated receptor (PPAR)-α, c-Jun N-terminal kinases (JNK), p38 mitogen-activated protein kinases (p38-MAPK), and nuclear factor kappa B (NF-κB). These receptors look to be largely synthesized in the brain of various mammals including humans, mice, and rats, and their expression has been reported in distinct brain parts such as the hypothalamus, pituitary, and subcortical and cortical neurons (Degawa-Yamauchi et al., 2003; Yamauchi et al., 2003; Fry et al., 2006; Hoyda et al., 2007; Psilopanagioti et al., 2009; Thundyil et al., 2010, 2012).

Thundyil et al. (2012) studied the expression of AdipoQ receptors in the CNS and demonstrated that AdipoQR1 is mostly released in the brainstem, hypothalamus, and the pituitary gland whereas AdipoQR2 seems to be mainly released in the cortex. Moreover, AdipoQR1 is intensely released in neurons and a smaller amount in astrocytes since AdipoQR2 is believed to be weakly expressed in neurons and astrocytes (Thundyil et al., 2012). The *AdipoQ* gene is broadly expressed in the hippocampus and the cortex. On the other hand, the T-cadherin receptor (CDH13), which is one of the receptors mediating AdipoQ activity, seems to be spatially and temporally expressed in various neuronal cells during axon development (Ranscht and Dours-Zimmermann, 1991). Besides, T-cadherin exhibits wide expression in the brain hippocampus, cerebral cortex, amygdala, and basal ganglia in the postnatal telencephalon of *Callithrix jacchus* (Matsunaga et al., 2013). CDH13 was further expressed by projection neurons within the main as well as accessory olfactory bulbs. Remarkably, AdipoQ deficiency is related to higher levels of inflammatory signals in serious illness or septic patients (Venkatesh et al., 2009; Hillenbrand et al., 2010, 2012). The expression of AdipoQR1 and AdipoQR2 was recently reported in primary human astrocytes and the U373 MG human glioblastoma astrocytoma cell line (Wan et al., 2014). It was further revealed that AdipoQ mediates pro-inflammatory signaling by rising interleukin (IL)-6, and monocyte chemoattractant protein 1 (MCP-1) notably *via* NF-κB, extracellular signal-regulated kinase (ERK)1/2, and p38-MAPK pathways in human astrocytes (Wan et al., 2014). Additionally, it has been suggested that AdipoQ may also regulate neuroinflammation by decreasing inflammatory cytokine expression through brain endothelial cells (Spranger et al., 2006).

A study reported the decreased expression of total AdipoQ and its high molecular weight oligomers in patients with common variable immunodeficiency (Pecoraro et al., 2017). A further study also revealed different expression patterns of AdipoQR1 and AdipoQR2 in the peripheral blood mononuclear cells of these patients (Polito et al., 2019).

## AdipoQ in Cognitive Function

Synapse dysfunction is the major pathological hallmark of cognitive impairment in AD (DeKosky and Scheff, 1990; Terry et al., 1991). Exciting data suggest that Aβ oligomers serve as neurotoxins that accumulate in the brain of AD patients leading to synaptic dysfunction and impaired synaptic plasticity as well as triggering synapse injury (Lambert et al., 1998; Ferreira and Klein, 2011; Forny-Germano et al., 2014). Oligomeric Aβ prevents long term potentiation (LTP) and thus stimulates long term depression (LTD) of hippocampal synapses both *in vitro* and *in vivo* (Wang et al., 2002; Shankar et al., 2008; Li et al., 2009; Jürgensen et al., 2011).

Several studies have proposed that AdipoQ signaling directly controls synaptic activity as well as plasticity, and maintaining and increasing cognitive activities in a variety of models. In anesthetized rats, intracerebroventricular administration of AdipoQ potentiates high-frequency stimulation (HFS)-mediated LTP and suppresses low-frequency stimulation (LFS)-mediated LTD (Pousti et al., 2018). Also, AdipoQ administration mediates a chemical LTP, irrespective of presynaptic incentive (Pousti et al., 2018). Treatment with a plant-derived homolog of AdipoQ (osmotin) modulated AdipoQ receptors ameliorated LTP damage and memory discrepancies in AD models (Shah et al., 2017). This activity seems to be induced by the Nogo66 receptor 1 and AdipoQR1 and comprises an outgrowth of neurite development and a rise of the dendritic spine as well as synapse density in the brain hippocampus (Zhang et al., 2016; Yoon et al., 2018).

AdipoQ-knockout mice show elevated excitability of the hippocampal dentate gyrus (DG) granule neurons together with reduced loss of contextual fear memory. AdipoQ and its analog drug AdipoRon, reestablished fear memory loss through AdipoQR2 stimulation and prevention of the excitability of DG neurons (Zhang et al., 2017). Elderly AdipoQ-knockout mice display decreased concentrations of synaptic proteins proposing synapse impairment, and poor scores in contextual fear conditioning and spatial memory tests (Ng et al., 2016). Interestingly, both osmotin and AdipoQ treatments improve learning as well as memory insufficiencies in AD animal models (Ali et al., 2015; Shah et al., 2017). Caloric constraints enhance circulating AdipoQ concentrations and increase cognition in mice perhaps through controlling the AMPK pathway in the mouse hippocampus (Ma et al., 2018). Furthermore, a clinical trial reported that patients with elevated AdipoQ concentrations exert better performances in a prolonged word memory test, advocating the idea that AdipoQ is a protective factor against cognitive failure suggesting a novel therapeutic approach in cognitive decline (Cezaretto et al., 2018).

## AdipoQ in Alzheimer’s Hallmarks

### Animal Studies

**Figure 1:**
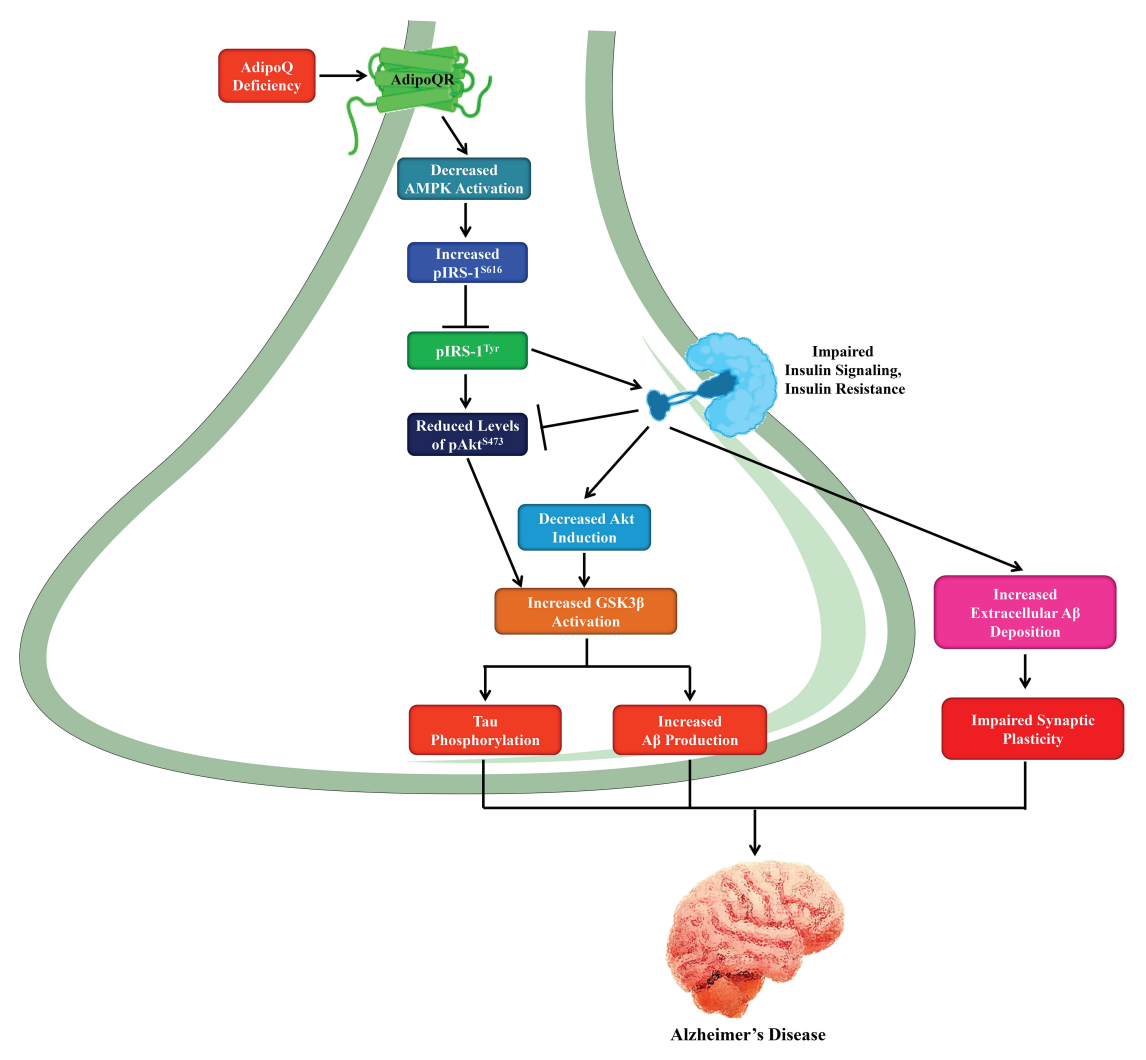
The role of AdipoQ deficiency in the pathogenesis of Alzheimer’s disease through AMPK inactivation. In neurons, AdipoQ deficiency leads to decreased AMPK activation and increased phosphorylation of IRS-1^S616^ that inhibits the pIRS-1^Tyr^ formation, and thus deactivates insulin signaling. On the other hand, insulin resistance is gradually developed and reduces pAkt^S473^ Levels. Thus, decreased Akt induction and increased GSK3β activation results in increased Aβ production and tau phosphorylation. Furthermore, insulin resistance exacerbates the extracellular Aβ deposition as well as impaired synaptic plasticity. AMPK, AMP-activated protein kinase; IRS, insulin receptor substrate; GSK3β, glycogen synthase kinase 3β; Akt, protein kinase B; Aβ, amyloid beta peptide.

Study suggests that insufficiency in AdipoQ signaling can mediate an AD-like symptom in mice models (Table 1; Ng et al., 2016). Aged AdipoQ-deficient mice recapitulate various features of AD pathology, such as increased levels of Aβ, synapse loss, neuroinflammation, tau hyperphosphorylation, neuronal cell death, and reduced insulin signaling. Significantly, aged AdipoQ deficient mice accomplished poor activity upon fear conditioning behavioral experiments and spatial memory (Ng et al., 2016). This study also indicated that chronic deficiency of AdipoQ in aged mice inactivates AMPK signaling thus causing insulin desensitization and provokes AD-like cognitive impairments as shown in Figure 1. The above interpretations were again validated by another analysis showing that gene-therapy mediated AdipoQR1 suppression further induces AD-like pathogenesis, which comprises dysfunction in the spatial memory as well as learning, insulin signaling deficiency, elevated concentrations of Aβ accretions and hyperphosphorylated tau, neuroinflammation as well as other neurodegenerative events (Kim et al., 2017). These analyses afford convincing reports for the implication of AdipoQ deficiency in the pathogenesis of AD. The nucleus basalis magnocellularis (NBM) is a well-known cholinergic nucleus in the basal forebrain that is disturbed rigorously in AD along with other neurodegenerative disorders. It has been reported that the dysfunction of NBM cholinergic neurons influences cholinergic loss and, more notably, is associated with clinical events of dementia (Arendt et al., 1983; Iraizoz et al., 1999; Liu et al., 2015a). Fascinatingly, NBM was reported to be an eminent site of AdipoQR1 expression (Psilopanagioti et al., 2009). Hence, it is likely that a lack of AdipoQ stimulates the development of AD by stimulating NBM degeneration.

**Table 1:** Several promising studies of the role of AdipoQ signaling in physiopathological processes associated with Alzheimer’s disease.

Therapeutic agent/target	Species/studied material/model(s)	Effects	Mechanism	References
AdipoQ	AdipoQ-deficient (Adipo^−/−^) mice, AdipoQ-haploinsufficient (Adipo^+/−^) mice	Neurotrophic effects on dendritic spine remodeling and neurogenesis in the dentate gyrus	Neurotrophic effects of AdipoQ	Zhang et al., 2016
AdipoQ	AdipoQR2^−/−^ mice, Adipo^−/−^ mice, AdipoQR1-floxed mice (AdipoQR1^flox/flox^)	Regulates contextual fear extinction and excitability of dentate gyrus neurons	AdipoQ/AdipoQR2 activation	Zhang et al., 2017
Osmotin	AdipoQ^−/−^ mice, B6.Cg-Tg (APPswe, PSENdE9)85Dbo/Mmjax AD model mice, C57BL/6J-Tg (NSE-APPsw) KLAR mice	Enhances the non-amyloidogenic pathway by activating the α-secretase gene and reverses the suppression of long-term potentiation	Modulates AdipoQR1/AMPK (AMP-activated protein kinase)/SIRT1 (Sirtuin 1)/SREBP2 (sterol regulatory element-binding protein 2) signaling pathway	Shah et al., 2017
AdipoQ	Adult male Wistar rats	Modulates synaptic plasticity in the hippocampal dentate gyrus	Enhances the long-term potentiation and suppresses long-term depression	Pousti et al., 2018
Osmotin	C57BL/6J-Tg (APPswe/PSEN1dE9) mice	Enhances neurite outgrowth and synaptic complexity	Modulates AdipoQR1/NgR1 signaling	Yoon et al., 2018
AdipoQ	AdipoQ-deficient mice	AdipoQ deficiency causes AD-like synapse loss and memory impairment	AMPK inactivation and cerebral insulin resistance	Ng et al., 2016
Osmotin	Male wild-type C57BL/6J mice	Prevents Aβ_42_-induced apoptosis, neurotoxicity, tau phosphorylation, and neurodegeneration	Modulates AdipoQR (adiponectin receptor)	Ali et al., 2015
AdipoQ	AdipoQR1^−/−^ mice	AdipoQ deficiency leads to memory dysfunction and AD-like pathologies	Suppresses AdipoQR1	Kim et al., 2017
AdipoQ (enriched environment-mediated)	AdipoQ^−/−^ mice	Reduces neuroinflammation and depressive-like behaviors	Activates anti-inflammatory state	Chabry et al., 2015
Globular AdipoQ	AdipoQ^−/−^ mice, AdipoQR2^−/−^ mice	Anti-inflammatory and anti-oxidant actions on microglia	Modulates AdipoQR1 (adiponectin receptor 1)/NF-κB (nuclear factor-κB) signaling Pathway	Nicolas et al., 2017
AdipoQ	5xFAD male transgenic mice, wild-type male C57BL/6 mice	Promotes anti-inflammatory responses on microglia	Modulating PPAR-γ (peroxisome proliferator-activated receptor-γ) signaling	Song et al., 2017
Recombinant C1q/TNF-related protein 9 (CTRP9)	BV2 microglial cells exposed to Aβ	Attenuates neuroinflammation, reduces brain edema, and improves neurological function	Activates AdipoQR1/AMPK/NF-κB signaling	Zhao et al., 2018
AdipoQ	Male Sprague-Dawley rats	Inhibits cerebral expression of myeloperoxidase and inflammatory mediators	Activates anti-inflammatory state and controlling NF-κB (p65)	Chen et al., 2009
AdipoQ	Male ICR mice	Protects hippocampal neurons	Preservation of the integrity of the blood-brain barrier (BBB)	Jeon et al., 2009
AdipoQ	Primary hippocampal cell cultures	Reduces the level of reactive oxygen species, attenuates apoptotic cell death, and also suppresses the caspase-3 activation	Modulates AMPK pathway	Qiu et al., 2011
AdipoQ	SH-SY5Y cell line	Reduces Aβ neurotoxicity	Activates AMPK and suppresses the NF-κB activation	Chan et al., 2012
Globular AdipoQ	Male C57BL/6 mice	Promotes antioxidant capacity	Inhibits NADPH oxidase-mediated oxidative damage	Song et al., 2013
Globular AdipoQ	Adult male C57BL/6mice	Improves neurological scores and reduces the infarct volumes	Modulates AdipoQ/AdipoQR expression	Song et al., 2015
AdipoQ (electroacupuncture pretreatment)	Male C57BL/6 mice	Exerts neuroprotective effects	AdipoQR1-mediated phosphorylation of glycogen synthase kinase-3β (GSK-3β)	Guo et al., 2015
CTRP3	Adult male Sprague-Dawley rats	Attenuates brain injury	Modulates AMPK-dependent pathway	Wang et al., 2016
CTRP3	Male Sprague-Dawley rats	Reduces cerebral edema, oxidative stress, and BBB damage and improves neurological functions as well as brain antioxidant enzymes	Modulates NADPH pathway	Yang et al., 2017
AdipoQ (caloric restriction-induced)	C57/BL mice	Improves hippocampus-dependent learning and memory	Modulates AMPK signaling pathway	Ma et al., 2018
AdipoQ (physical exercise-induced)	Male WT C57BL/6J mice, *AdipoQ*^−/−^ mice	Promotes hippocampal neurogenesis and depression	Activates the ADNR1/AMPK signaling pathways	Yaua et al., 2014
AdipoQR1 and AdipoQR2	Wild-type C57BL/6J mice, APP(swe)/Presen(e9d)1 (AD) mice	AdipoRs are less sensitive to stress	Modulates AdipoQR expression	Várhelyi et al., 2017
AdipoQ supplements	Streptozotocin injected male Sprague-Dawley rats	Reduces tau hyperphosphorylation and increases dendritic branches number and mushroom percentage	Activates the PI3K (phosphatidylinositol 3-kinase)/Akt (protein kinase B)/GSK-3β signaling pathway	Xu et al., 2018
AdipoQ	Middle-aged-to-elderly community-dwelling persons	Deficiency of plasma AdipoQ was associated with mild cognitive impairment (MCI) in men	-	Kamogawa et al., 2010
AdipoQ	Non-diabetic participants of ELSA-Brasil	Enhances cognitive performance	Neuroprotective effects of AdipoQ	Cezaretto et al., 2018
AdipoQ	MCI and Alzheimer’s disease (AD) patients	Plasma AdipoQ was considerably higher in MCI and AD, while cerebrospinal fluid (CSF) AdipoQ was meaningfully higher in MCI compared to controls	-	Une et al., 2011
AdipoQ	Women with a median age of 76years (prospective cohort study)	Increased plasma AdipoQ levels serve as a risk factor (independent) for the progress of all-cause dementia and AD	-	Van Himbergen et al., 2012
AdipoQ	MCI and AD patients	Deficiency of AdipoQ was linked to cognitive dysfunction (however additional cognitive decline/dementia was not predicted in this cohort)	-	Teixeira et al., 2013
AdipoQ	Probable AD patients	Alteration of serum AdipoQ levels implies amyloid pathology, neurodegeneration, and hypometabolism of glucose	-	Khemka et al., 2014
AdipoQ	Type 2 diabetes patients	AdipoQ deficiency correlates with AD-like brain changes	-	García-Casares et al., 2016
AdipoQ (Baduanjin Qigong exercise-induced)	Female participants with chronic fatigue syndrome-like illness	Reduces symptoms of depression	Modulates AdipoQ levels	Chan et al., 2017

Conversely, loss in AdipoQ activity was also detected in amyloid-induced AD prototypes, while triggering the AdipoQ signaling pathway declines AD-like phenotypes. APP/PS1 mice displaying alterations in the levels of AdipoQ receptor expression seemed less reactive to a stress-mediating mice model as compared to control mice (Várhelyi et al., 2017). In an identical paradigm, osmotin improved AD-like phenotypes, for example, Aβ formation and deposition, synaptic loss and deficient LTP, and cognitive decline and memory loss. AdipoQR1 silencing eliminates the beneficial effects of osmotin and additionally aggravates brain pathology in AD-mice (Shah et al., 2017). Osmotin was also found to diminish Aβ accumulation in the cultured cells of SH-SY5Y human neuroblastoma overexpressing APP. Osmotin actions are induced by stimulation of the AMP kinase, an enzyme downregulated by oligomeric Aβ in hippocampal neurons (Seixas da Silva et al., 2017). An intracerebroventricular dose of AdipoQ recovered cognitive decline and reduced glycogen synthase kinase 3β (GSK3β)-induced tau hyperphosphorylation in AD-related areas in a rat paradigm of streptozotocin-mediated brain pathology (Xu et al., 2018). These reports propose the stimulation of the AdipoQ signaling pathway, predominantly *via* AdipoQR1, as a novel therapeutic target in AD (Ng and Chan, 2017). Considering this, chronic administration of a widely used anti-AD acetylcholinesterase antagonist (i.e., donepezil) was found to elevate serum concentrations of AdipoQ (Pákáski et al., 2014). Furthermore, thiazolidinediones (TZDs) including pioglitazone and rosiglitazone, as well as PPARγ agonists (i.e., used for type 2 diabetes mellitus; Saltiel and Olefsky, 1996; Malinowski and Bolesta, 2000; Hong et al., 2018) were just repurposed to combat AD. The insulin-sensitizing function of TZDs is in part induced by triggering peripheral AdipoQ receptors and *AdipoQ* gene expression (Yu et al., 2002; Tsuchida et al., 2005; Nie and Li, 2017). Consequently, it is feasible that AdipoQ exerts part of the experimental benefits of TZDs and donepezil.

AdipoRon, an AdipoQ receptor agonist (Okada-Iwabu et al., 2013), has been shown to regulate hippocampal synaptic transmission and to accelerate fear memory loss in rodents (Zhang et al., 2017). Also, AdipoRon was shown to control dopaminergic neuronal activity in the ventral-tegmental site (Sun et al., 2019) and act as a metabolic and antidepressant controller in a mouse model of depression (Nicolas et al., 2018). Notably, central AdipoRon activities were achieved by peripheral inoculation, and it has been found that this drug passes the blood-brain barrier (BBB) to trigger AdipoQ receptors in the brain. It would be exciting to further examine the effects of this AdipoQ-drug analog with translational potential in AD models.

### Human Studies

In humans, analyses relating AdipoQ concentrations to AD are debatable. Improved baseline AdipoQ concentrations in plasma were found to be involved with an elevated risk of developing AD and other forms of dementia in women, but not men (Table 1; van Himbergen et al., 2012). Moreover, increased AdipoQ levels were revealed in the cerebrospinal fluid (CSF) and plasma of individuals with MCI as well as sporadic AD (Une et al., 2011; Khemka et al., 2014), while plasma concentrations of AdipoQ was positively associated with dementia. The relationship between AD and increased blood AdipoQ levels has been advocated by meta-analysis (Ma et al., 2016). Though the afore-mentioned reports suggest elevated AdipoQ concentrations to be related to Alzheimer’s dementia, conflicting effects have also been described. Data analysis has shown decreased concentrations of AdipoQ in MCI and AD individuals. Furthermore, based on the AdipoQ levels, one is not able to foresee the development of cognitive decline from normal conditions to MCI and from MCI to AD, respectively (Teixeira et al., 2013). Another report also revealed that reduced concentrations of AdipoQ in plasma were involved with MCI, although this link was identified in males, but not in females (Kamogawa et al., 2010). From these experiments, it has been found that decreased levels of plasma AdipoQ in diabetic patients is associated with a lower volume of gray-matter and lower glucose uptake in the temporal region of the brain, comparable to what is perceived in AD (García-Casares et al., 2016). Furthermore, another study reveals that AdipoQ concentrations in blood are elevated but the lower levels are reported in CSF of MCI and AD individuals. Existing studies concerning a link between AdipoQ concentrations in blood and CSF to MCI and AD are inconsistent and questionable. Hence, more analyses are needed to explore the precise impact of AdipoQ. Several reports have shown that the relationship between dementia and AdipoQ levels might be sex-dependent (Kamogawa et al., 2010). This might be particularly significant in the case of AD, where a significant sexual dichotomy has been reported with females being at a considerably increased risk of developing AD than males (Uddin et al., 2020c).

## AdipoQ in Neuroinflammatory Signaling of Alzheimer’s Disease

The brain of AD patients exerts a prolonged state of low-level inflammation, which is induced by the activation of microglial cells that leads to the secretion of several inflammatory cytokines such as interleukin (IL)-6, tumor necrosis factor-α (TNFα), and IL-1β (Bamberger et al., 2003; Lourenco et al., 2013; Heneka et al., 2015; Hansen et al., 2018; Uddin et al., 2020b). These cytokines induce various harmful incidents in the AD brain such as endoplasmic reticulum stress, neuronal insulin resistance, synaptotoxicity, and finally neurodegeneration (Bomfim et al., 2012; Lourenco et al., 2013; Rizzo et al., 2018).

AdipoQ-deficient mice possess a variety of clinical features in the AD brain, such as decreased concentrations of synaptic proteins, insulin resistance, and the existence of neuroinflammatory markers including astrogliosis, microgliosis, and higher concentrations of the inflammatory cytokines such as IL-1β and TNFα (Ng et al., 2016). Environmental upgrading like housing situations that stimulate social interactions, cognitive engagement, and physical activity, has been found to increase cognitive activities in AD mice. These results are at least in part owing to the alteration of the microglial effect to the insults of neurotoxic Aβ oligomers (Xu et al., 2016; Vieira and Beckman, 2017). Recently, it has been shown that beneficial outcomes of environmental upgrading to the brain are induced by AdipoQ including the advancement of an anti-inflammatory state of microglia with the lower secretion of inflammatory cytokines (Chabry et al., 2015; Nicolas et al., 2015). It has also been reported that globular AdipoQ prevents the microglia inflammatory profile directly *in vitro* and *in vivo* (Nicolas et al., 2017) *via* a mechanism involving NF-κB and AdipoQR1. AdipoQ also regulates the *in vitro* activation of microglia under Aβ toxicity through PPARγ stimulation (Song et al., 2017). Another report suggesting the anti-inflammatory effects of AdipoQ in the CNS shows that an AdipoQR1 agonist (i.e., CTRP9) reduces neuroinflammatory effects in an *in vivo* mouse model of intracerebral hemorrhage *via* AdipoQR1/AMPK/NF-κB signaling pathways (Zhao et al., 2018). Besides, AdipoRon usage was found to block the recruitment of macrophages in an experimental model of spinal cord damage (Zhou et al., 2019).

AdipoQ was reported to prevent the pro-inflammatory response, remarkably by blocking IL-6 discharge from BBB endothelial cells (Spranger et al., 2006). It has been shown that AdipoQ regulates inflammatory signaling indirectly through the BBB by adversely modulating TNFα and IL-6 release. Laboratory analysis of hippocampal neurons has indicated that AdipoQ mediates neuroprotective effects *via* the AMPK signaling pathway (Qiu et al., 2011). Such effects are additionally supported by experiments demonstrating that AdipoQ knockout mice show enlarged brain injuries and increased neurological deficits after ischemia-reperfusion compared with wild-type mice (Nishimura et al., 2008). These studies provide evidence that the neuroprotective effect of AdipoQ is induced *via* an endothelial nitric oxide synthase (eNOS)-reliant mechanism (Nishimura et al., 2008). Thus the neuroprotective role of AdipoQ takes part in the regulation of brain inflammation; it was proposed that the lack of AdipoQ in obesity might induce neuroinflammation leading to AD and other dementias.

## AdipoQ in Insulin Signaling of Alzheimer’s Disease

Neuronal insulin signaling plays an important role in memory and synaptic plasticity, mostly by controlling glutamate receptor transport (Beattie et al., 2000; Man et al., 2000; Skeberdis et al., 2001; Zhao et al., 2004). Defective insulin signaling is well-reported both in AD individuals and in a number of AD animal models (Steen et al., 2005; Bomfim et al., 2012; Talbot et al., 2012). Impaired insulin signaling is responsible for the neuronal loss and cognitive decline in AD (Ferreira et al., 2014; Vieira et al., 2018). The synaptotoxicity of Aβ is associated with a loss of insulin receptor activity *in vivo* and *in vitro* and can be inhibited by using insulin itself and by insulin-sensitizing medications (De Felice et al., 2009; Bomfim et al., 2012; Batista et al., 2018). These findings prompted numerous groups to investigate the activity of various types of anti-diabetic therapies in AD prototypes, and progressive preclinical outcomes triggered researchers to complete human clinical tests (De Felice, 2013; Yarchoan and Arnold, 2014; de la Monte, 2017). In this regard, attention has been focused on the insulin-sensitizing activities of AdipoQ to rectify the abnormal insulin signaling in AD.

A diminished brain insulin signaling cascade was detected, along with various other AD-resembling pathological symptoms, in AdipoQ-deficient mice as well as in AdipoQR1-knockout mice (Ng et al., 2016; Kim et al., 2017). In contrast, AdipoQ was found to enhance insulin susceptibility in the SH-SY5Y neuroblastoma cell line exhibiting insulin resistance, *via* AdipoQR1 initiation of AMPK (Ng et al., 2016). These studies reveal that AdipoQ possesses the possibility to reestablish neuronal insulin signaling, with potential therapeutic effects for AD and also other neurodegenerative disorders (Bloemer et al., 2018). Nevertheless, more translational analyses using AD models are needed to authenticate the neuroprotective role of AdipoQ as therapeutic interventions to halt brain insulin resistance in AD.

Other studies suggested that insulin resistance (Capurso and Capurso, 2012; Johnson and Olefsky, 2013; Medina-Urrutia et al., 2015) is associated with an excess of adiposity induced by high free fatty acid (Perseghin et al., 2003) and lower levels of plasma AdipoQ (Arita et al., 2012). Furthermore, acute lowering of free fatty acid is linked to decreased AdipoQ levels (Bernstein et al., 2004). A study has revealed that the altered plasma status of both docosahexaenoic acid and other fatty acids unrelated to docosahexaenoic acid are linked to AD (Cunnane et al., 2012).

## AdipoQ Paradox in Alzheimer’s Disease

AdipoQ is a well-known neuroprotective agent against various cytotoxicities induced by Aβ as well as MPP^+^ *in vitro* (Jung et al., 2006; Chan et al., 2012). In mice brains, AdipoQ is protected from kainic acid-mediated excitotoxicity in the hippocampus (Jeon et al., 2009). Particularly, AdipoQ may control neurogenesis. Regarding this concept, AdipoQ has been reported to incite proliferation of mature hippocampal neural stem/progenitor cells *via* signaling pathways including GSK3β/p38-MAPK/β-catenin (Zhang et al., 2011). Moreover, it has been shown that physical exercise triggers hippocampal neurogenesis, facilitated by AdipoQ (Yaua et al., 2014). Furthermore, AdipoQ was found to be neurotrophic for spinogenesis and dendritic arborization in the DG in mice brains (Zhang et al., 2016). Several neuropathological characteristics, e.g., compromised motor activity and protein accumulation were improved by AdipoQ treatment in mouse experiments of α-synucleinopathies (Sekiyama et al., 2014). It was previously demonstrated that elderly AdipoQ-defective mice initiated an AD-like pathology linked with dysregulation of insulin receptor signaling (Ng et al., 2016).

The outcome of a cohort study performed by the Mayo Clinic Study of Aging indicated that elevated levels of plasma AdipoQ were interrelated with imaging data for cortical and hippocampal volumes, cognitive declines, and positron emission tomography (Wennberg et al., 2016). Therefore, the obtained results advocate that higher AdipoQ predicts cognitive decline and neurodegeneration in aging (Wennberg et al., 2016). Amazingly, these experimental outcomes were significant in females but not in males, which is consistent with the analyses of the Framingham Heart Study (van Himbergen et al., 2012). Consistent with the prediction that AdipoQ might be included in neurodegenerative pathogenesis, histopathological studies of an autopsy of an AD brain demonstrated that AdipoQ was segregated into the neurofibrillary tangles by tau (Waragai et al., 2016). AdipoQ also co-localized with Lewy bodies in the brain of dementia patients (Sekiyama et al., 2014). Along with plasma data regarding AdipoQ, the overexpression of AdipoQ might be associated with the progression of neurodegenerative disorders like AD. In a retrospective cohort study, Qizilbash et al. (2015) examined the involvement of BMI in developing dementia. Both underweight middle-aged and elderly people displayed an elevated probability of developing dementia within the next two decades. The presence of dementia started to decline with an increase in BMI, with highly obese patients (BMI >40kg/m^2^) developing a 29% reduction (95% CI: 22-36) in the possibility of developing dementia than those of healthy-weight individuals.

**Figure 2:**
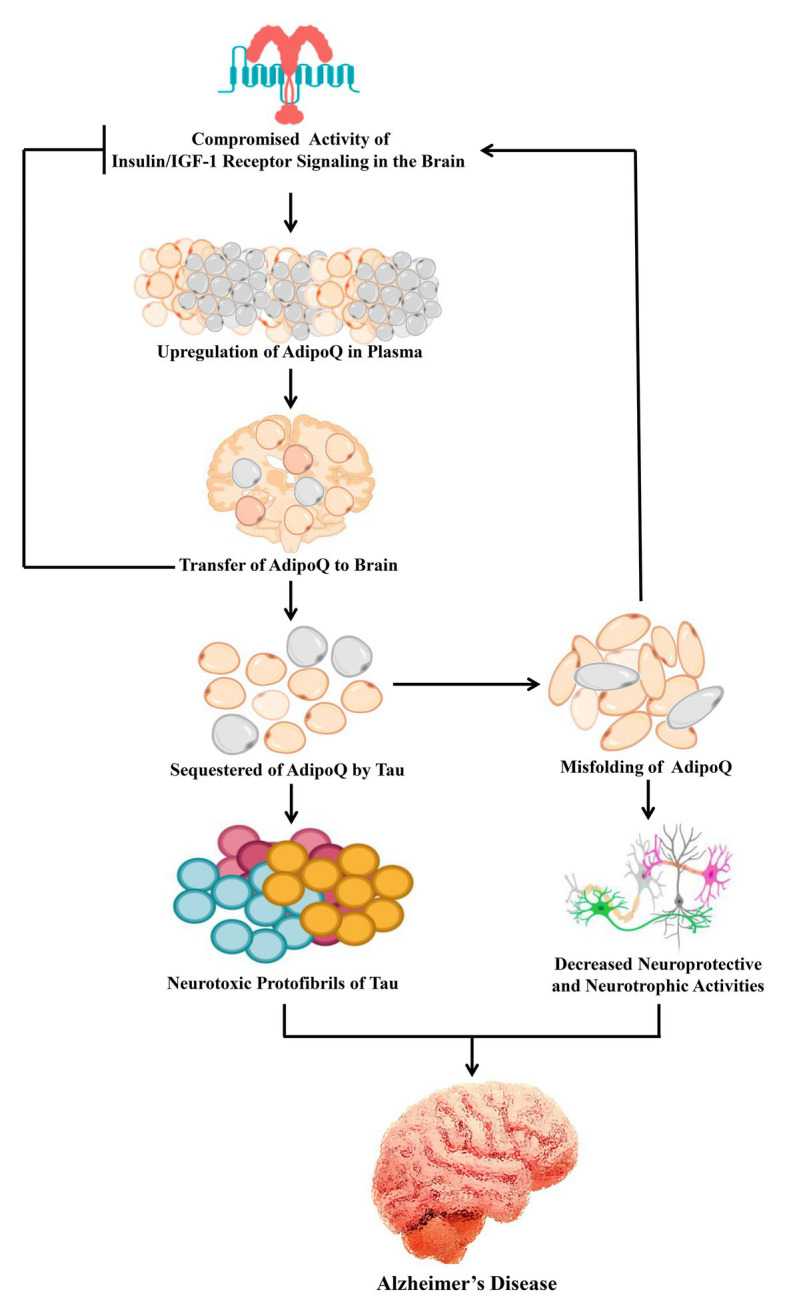
Possible mechanism of AdipoQ signaling in Alzheimer’s disease. In the brain, insulin resistance is a common feature of Alzheimer’s disease. AdipoQ is upregulated to compensate for the compromised activity of insulin/insulin/IGF-1 receptor signaling. As a result the plasma levels of AdipoQ are transferred to the brain and improve the compromised insulin signaling. Furthermore, AdipoQ is sequestered by tau which leads to the formation of neurotoxic protein aggregation. On the other hand, such sequestration of AdipoQ is associated with its misfolding which leads to the downregulation of insulin signaling as well as decreased neuroprotective and neurotrophic activities. IGF-1, insulin-like growth factor 1.

Until now, the mode of hyperadiponectinemia in AD has not been clear. Hyperadiponectinemia in AD is expected to be a compensatory response to the lower efficiency of the insulin/insulin-like growth factor 1 (IGF-1) receptor signaling cascade during neurodegeneration (Waragai et al., 2017). As the disease progresses, AdipoQ might be increased as well as sequestered by tau, which ultimately leads to the aggregation of neurotoxic proteins in the AD brain (Figure 2; Waragai et al., 2016, 2017). A different alternative mechanism is that the misfolding of AdipoQ might downregulate the insulin/AdipoQ signal transduction network causing the reduction of neuroprotective and neurotrophic functions (Waragai et al., 2017). Therefore, it is expected that a mutation of AdipoQ may trigger synaptic dysfunction and neuronal death in AD.

## Conclusion

Obesity in middle-aged people might be considered as a critical factor in the development and progression of AD in the elderly. The higher possibility of people with obesity to develop AD reveals the ability of adipose tissue to connect with the brain and influence its activity. AdipoQ signaling has been found to interact with a variety of neuropathological incidents such as Aβ formation and deposition, tau hyperphosphorylation, neuroinflammation, insulin resistance, cognitive damage, and synaptic loss. The phenotypes of AdipoQ or AdipoQ receptor-knockout mice recapitulates most of the AD neuropathological features. Thus, abnormal AdipoQ signaling may induce obesity-mediated harmful effects on the CNS and increase the risk of cognitive impairment and AD. Notably, in the brain, restoring typical AdipoQ signaling might establish constructive, disease-modifying therapeutics to combat AD.

## Author Contributions

MU conceived the original, designed the outlines of the study, and prepared the figures for the manuscript. MU, MR, and MS wrote the draft of the manuscript. PJ edited the whole manuscript. MSA, GA, MB-J, SM, MA-D, MFA, and AS revised and improved the draft. All authors have read and approved the final manuscript.

### Conflict of Interest

The authors declare that the research was conducted in the absence of any commercial or financial relationships that could be construed as a potential conflict of interest.

## Abbreviations

AD: Alzheimer’s disease
AdipoQ: adiponectin
Aβ: amyloid-beta
APP: amyloid precursor protein
BMI: body mass index
MCI: mild cognitive impairment
AdipoQR1: AdipoQ receptor 1
AdipoQR2: AdipoQ receptor 2
